# Supplementary material for: Altitude-dependent agro-ecologies impact the microbiome diversity of scavenging indigenous chicken in Ethiopia
Source: Microbiome. 2024 Jul 23;12:138. doi: 10.1186/s40168-024-01847-4 (PMC11267795; doi:10.1186/s40168-024-01847-4)
Supplement: Supplementary file 2 — Additional file 1. Feed of indigenous Ethiopian chickens. [file 40168_2024_1847_MOESM1_ESM.docx]

**Feed of indigenous Ethiopian chickens**

Indigenous Ethiopian chickens primarily obtain sustenance by scavenging leftover food, insects, grains and vegetation [1-4]. In addition to what they scavenge from their environment, farmers supplement their diets with grains and kitchen waste. These may consist of finger millet (*Eleusine coracana*), wheat, barley, sorghum, and maize, depending on the grain that is accessible within the various agro-ecologies. For example, owners of chickens in highland locations in the extreme north (such as Negasi Amba, Alfamidir, Batambe, Gafera, Surta, and Amesha Shinkuri) supplement their feed with wheat and barley. On the other hand, chicken farmers in regions of moderate elevation augment their crop with maize, finger millet (like Ashuda and Dikuli), and occasionally sorghum (like Arabo, Adane, Tsion Teguaz, Gijet, Metkilimat, Mihiquan, and Hadush Adi). Sorghum is supplied as a supplement by chicken farmers in Kumato and Gesses, two dry and semiarid lowland regions. Even though intentional teff (*Eragrostis teff*) supplementation is uncommon in Ethiopia, indigenous chickens also scavenge leftover teff grains from that which spills on the ground during processing for milling.

Injera and kocho are examples of foods that are frequently supplemented in many chicken-raising families. Injera is a fermented flatbread made from teff to which smallholder chicken producers in Ethiopia add finger millet. Only southern Ethiopia, where Ensete (*Ensete ventricosum*/false banana) is frequently cropped, receives kocho supplementation. For many people in Ethiopia, kocho is a staple food made from chopped and grated ensete pulp. Along with bread and cereal, additional non-traditional supplements include vegetables, such as tomatoes and onions. On rare occasions, farmers in metropolitan and peri-urban areas supply industrial byproducts as supplements, such as oilseed cakes (also known as Noug seed cakes), wheat bran, and rice bran.

**References**

1. Mengesha M. Feed Resources and Chicken Production in Ethiopia. J World's Poult Sci. 2012;68(3):491-502. doi:10.1017/S0043933912000591.

2. Mekonnen H, Mulatu D, Kelay B, Berhan T. Assessment of the Nutritional Status of Indigenous Scavenging Chickens in Ada’a district, Ethiopia. Trop Anim Health Prod. 2010;42(1):123-30. doi:10.1007/s11250-009-9395-7.

3. Admasu S, Solomon D, Meseret M. Poultry Feed Resources and Chemical Composition of Crop Content of Scavenging Indigenous Chicken. J Anim Feed Res. 2019;9(6):247-55. doi:10.36380/scil.2019.ojafr34

4. Bekele B. Reviewing Locally Available Chicken Feed Resources at Different Agro-Ecologies (High, Mid and Low Land), Formulating According to Growth Stage (Growers and Layers) of Chicken and its Average Price in SNNPR. Journal of Aquaculture & Livestock Production. 2020;1(1):1-6. doi:10.47363/JALP/2020(1)102.
