# Supplementary material for: Altitude-dependent agro-ecologies impact the microbiome diversity of scavenging indigenous chicken in Ethiopia
Source: Microbiome. 2024 Jul 23;12:138. doi: 10.1186/s40168-024-01847-4 (PMC11267795; doi:10.1186/s40168-024-01847-4)
Supplement: Supplementary file 4 — Additional file 3: Fig. S1. Construction of a chicken caecal reference gene catalogue. The 3,629,587 non-redundant genes contained in the catalogue represent the metagenomes of 240 chicken caecal contents samples. Non-redundant genes were assigned to different taxon levels based on their last common ancestor in the Uniprot database (version 2019_03). Fig. S2. Description of the gene catalogue constructed from the caecal microbiota of Ethiopian indigenous chickens. A) Rarefaction analysis of the number of non-redundant genes vs sampling number. B-E) Break down of the taxa identified in the Ethiopian chicken caecal microbial gene catalogue, by Kingdom. Fig. S3. Genome statistics of high-quality, non-redundant strain-level (A-E) and species-level (F-J) metagenome-assembled genomes, as defined by CheckM. A and F: Completeness and contamination – dashed red lines indicate cutoffs for defining genomes as high-quality. B and G: Percentage GC content. C and H: log10 number of contigs per genome. D and I: Genome size (mb). E and J: log10 N50 of contigs. Fig. S4. The proportions of annotated read after mapping raw sequencing reads to the non-redundant gene catalogue (A) and MAGs (B). Fig. S5. Violin plot showing the number of CAZyme genes per strain-level MAG by dataset. A) Total unique CAZyme families. B) Total unique Glycoside Hydrolases (GH) families. C) Total CAZyme genes. D) Total GH genes. Fig. S6. Heatmap showing the percentage of species-level MAGs within genera with particular metabolic pathways. Genera were clustered at 40% AAI using the output from comparem. The uniqueness of genera in comparison to previous datasets is indicated. Genus-level clusters were not unique based on GTDB if any MAGs within that cluster were assigned a taxonomy at the genus level. MAGs were defined as not unique when compared to previous chicken microbial datasets (“not_unique_drep”) if they clustered at 99% (strain) or 95% (species) ANI with any non-scavenging chickens (NSC) microbial gen [file 40168_2024_1847_MOESM3_ESM.docx]

**Figure S1:**


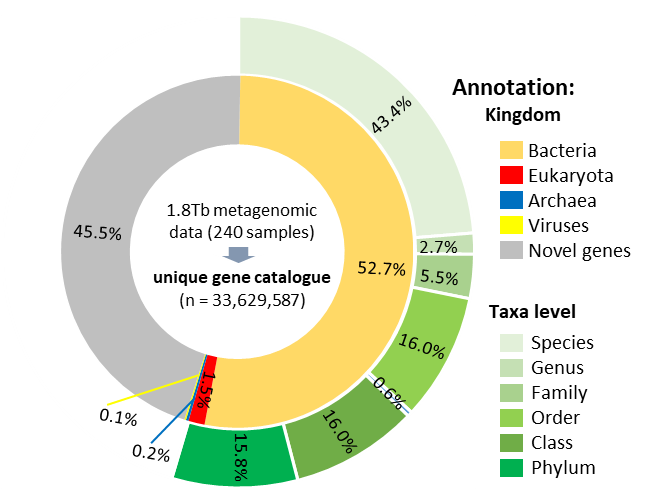


**Construction of a chicken caecal reference gene catalogue. The 3,629,587 non-redundant genes contained in the catalogue represent the metagenomes of 240 chicken caecal contents samples. Non-redundant genes were assigned to different taxon levels based on their last common ancestor in the Uniprot database (version 2019_03).**

**Figure S2:**


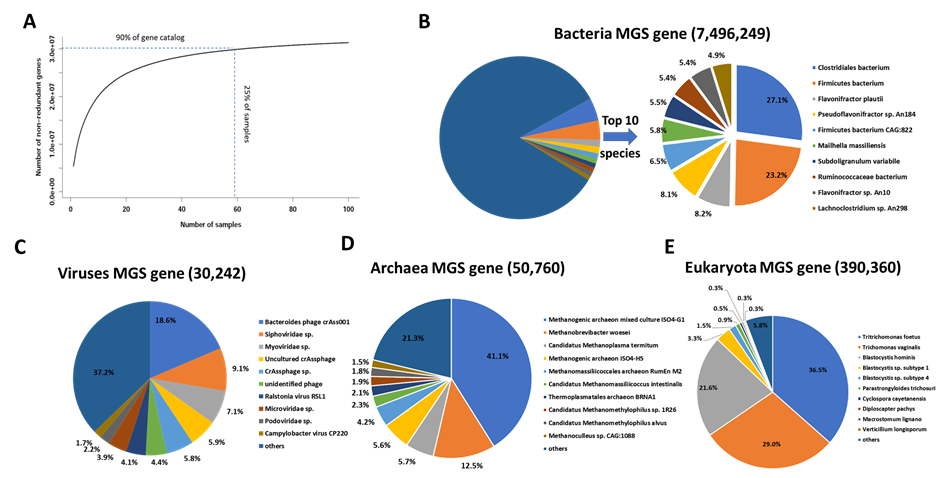


**Description of the gene catalogue constructed from the caecal microbiota of Ethiopian indigenous chickens. A) Rarefaction analysis of the number of non-redundant genes vs sampling number. B-E) Break down of the taxa identified in the Ethiopian chicken caecal microbial gene catalogue, by Kingdom.**

**Figure S3:**


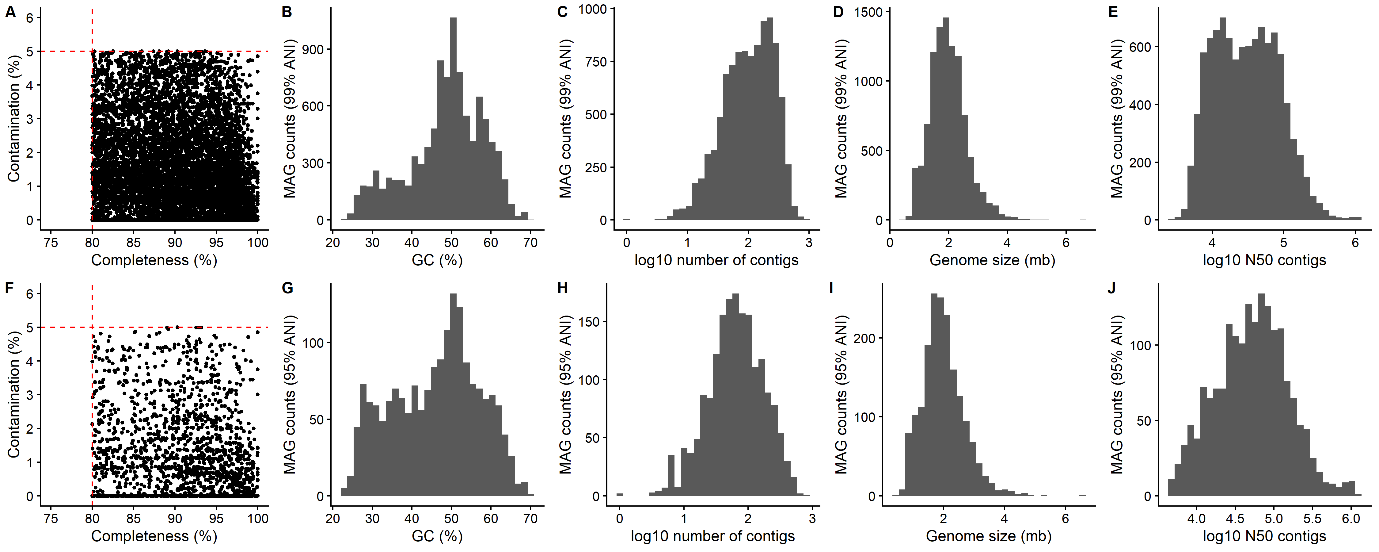


**Genome statistics of high-quality, non-redundant strain-level (A-E) and species-level (F-J) metagenome assembled genomes, as defined by CheckM. A and F: Completeness and contamination – dashed red lines indicate cutoffs for defining genomes as high-quality. B and G: Percentage GC content. C and H: log10 number of contigs per genome. D and I: Genome size (mb). E and J: log10 N50 of contigs.**


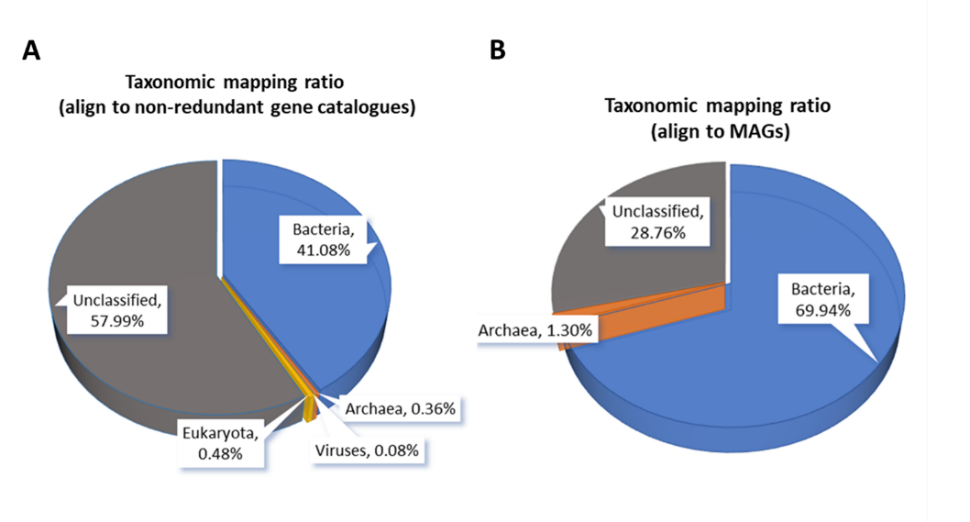
**Fig S4:**

**The proportions of anotated read after mapping raw sequencing reads to the non-redundant gene catalogue (A) and MAGs (B).**

**Figure S5:**

**
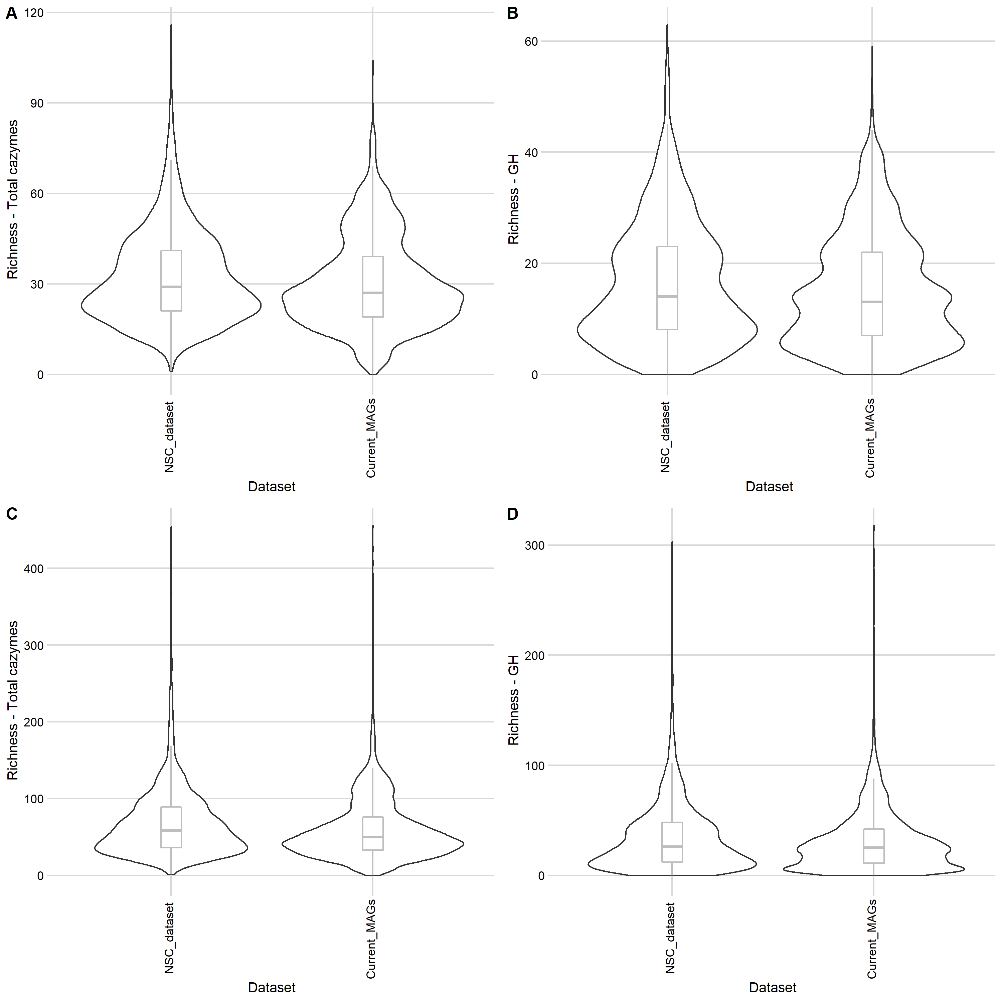
**

**Violin plot showing number of CAZyme genes per strain-level MAG by dataset. A) Total unique CAZyme families. B) Total unique Glycoside Hydrolases (GH) families. C) Total CAZyme genes. D) Total GH genes.**

**Figure S6: Heatmap showing the percentage of species-level MAGs within genera with particular metabolic pathways. Genera were clustered at 40% AAI using the output from comparem. The uniqueness of genera in comparison to previous datasets are indicated. Genus-level clusters were not unique based on GTDB if any MAGs within that cluster were assigned a taxonomy at genus level. MAGs were defined as not unique when compared to previous chicken microbial datasets (“not_unique_drep”) if they clustered at 99% (strain) or 95% (species) ANI with any non-scavenging chickens (NSC) microbial genome. Genera were defined as not unique when compared to previous chicken microbial datasets (not_unique_comparem) if they clustered at 60% AAI with any NSC microbial genome.**

**Figure S7:**

**
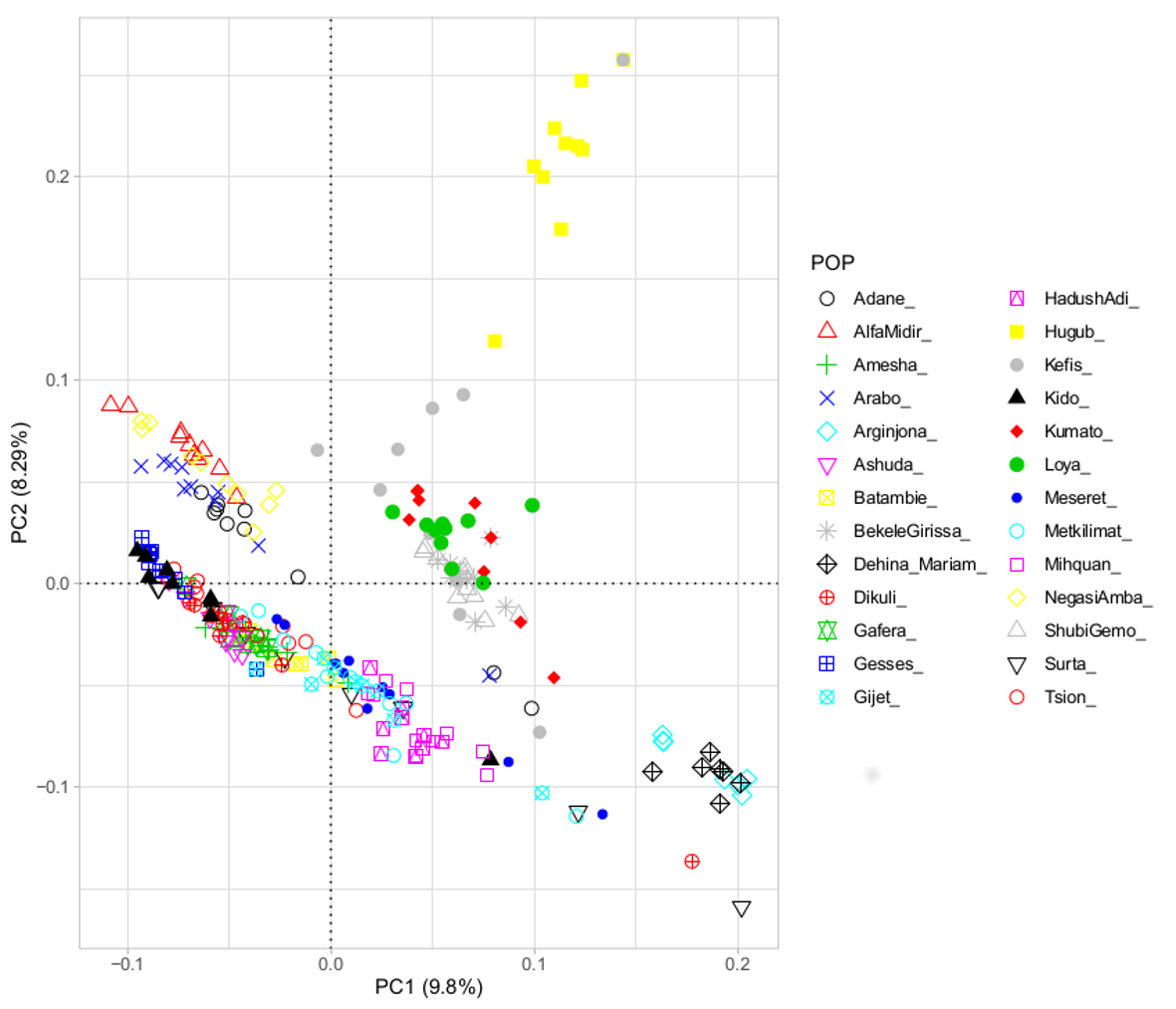
**

**Principle coordinate analysis showing the clustering of samples by autosomal SNPs. Samples are labelled by the region in which the sample was collected.**

**Figure S8:**


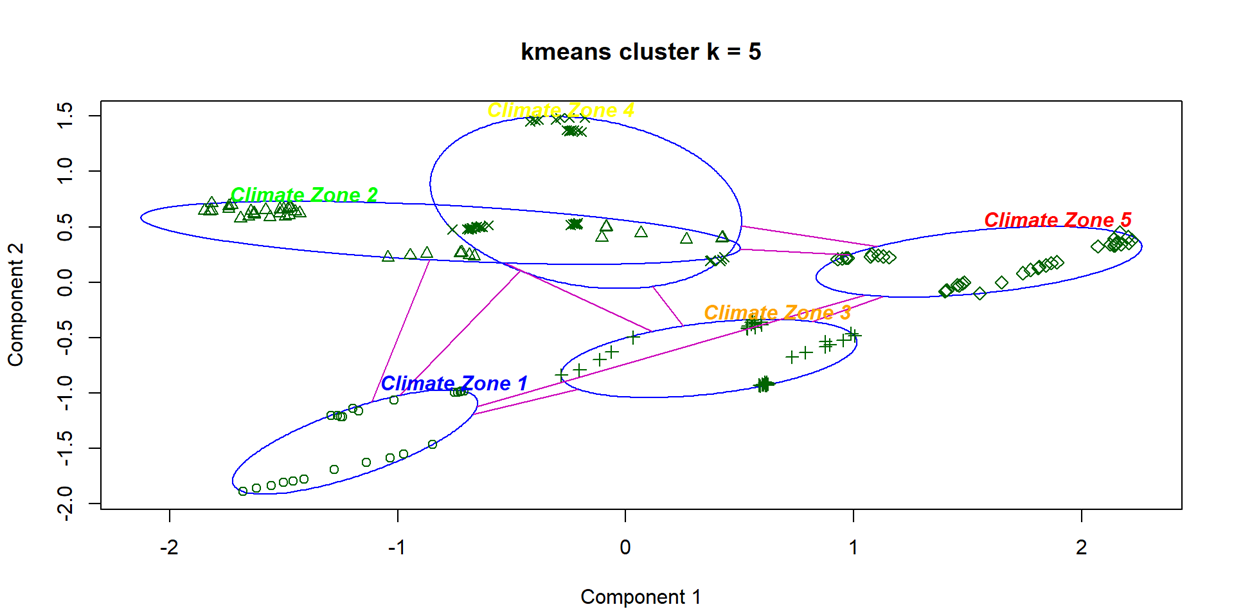


**Five climate zones, clustered using Kmeans according to annual temperature, annual precipitation and precipitation of the driest quarter of the sampling location between 1970 - 2000. Components 1 and 2 explain 85.1% of sampling site variation.**

**Table S1: Diversity of CAZymes in strain level MAGs**

| CAZyme module | Abbreviation | Module function | Families present in strain level MAGs |
| --- | --- | --- | --- |
| Auxiliary activities | AA | Redox enzymes acting in conjunction with CAZymes | 7 |
| Carbohydrate esterases | CE | Hydrolysis of carbohydrate esters | 16 |
| Carbohydrate-binding modules | CBM | Adhesion to carbohydrates | 50 |
| Glycoside hydrolases | GH | Hydrolysis/rearrangement of glycosidic bonds | 126 |
| Glycosyltransferases | GT | Formation of glycosidic bonds | 62 |
| Polysaccharide lyases | PL | Non-hydrolytic cleavage of glycosidic bonds | 24 |
